# Semantic dementia: a complex and culturally influenced presentation

**DOI:** 10.1192/bjb.2022.100

**Published:** 2024-02

**Authors:** Richard H. Cole, Camilla N. Clark, Norman A. Poole

**Affiliations:** 1 Camden and Islington NHS Foundation Trust, London, UK and Institute of Psychiatry, Psychology & Neuroscience, King's College London, UK; 2 St George's University of London, London, UK; 3 South West London and St George's Mental Health NHS Trust, London, UK

**Keywords:** Psychotic disorders, semantic dementia, frontotemporal dementia, clinical neurology, hyperreligiosity

## Abstract

The variants of frontotemporal dementia (FTD) require careful differentiation from primary psychiatric disorders as the neuropsychiatric manifestations can overshadow the unique cognitive deficits. The language variants of FTD are less readily recognised by trainees despite making up around 43% of cases.^1^ This educational article presents an anonymised case of one of the language variants: semantic dementia. The cognitive deficits and neuropsychiatric manifestations (delusions and hyperreligiosity) are explored in terms of aetiology and management. By the end of the article, readers should be able to differentiate FTD from Alzheimer's disease, understand the principles of management and associated risks, and develop a multifaceted approach to hyperreligiosity in dementia.

Informed consent was obtained from the patient's family for publication of their case details, as the patient has since passed away.

## Clinical scenario

*Doctor's perspective:* You are a core trainee working in the older adults’ community mental health clinic. A 69-year-old West African woman is referred by her general practitioner (GP) for ‘responding to unseen stimuli’ and ‘increasingly religious thinking’. She has a history of hypertension and severe depression in her 40s. Her GP reported her bloods as unremarkable.

*Patient's account:* She felt well other than some problems with her memory. She was able to recall more recent day-to-day events, but she relied on her son to recount autobiographical memories of her early life. She emphasised repeatedly that she is a devout follower of God, that the Lord is powerful and that she was being ‘attacked’ by demons and spirits. She spoke about having ‘the gift’ of being able to raise the dead and that her GP had poisoned her blood test.

*Collateral:* Her son reported that despite being religious (Pentecostal) lifelong, the intense expression of her religious belief was new. This change occurred insidiously over several years and 2 years ago he had asked the GP to alert mental health services. The patient had moved in with her son as she was no longer coping at home. She had incorrectly cut a wire while changing a plug, struggled with using kitchen appliances, limited her dietary repertoire and had started talking to strangers about God. She had also begun ‘speaking in tongues’ and was fixated on trying to print money to donate to those in need.

### The cognitive examination

The patient was partially oriented to time. Although she could not name the season, she correctly identified that summer had recently ended. She exhibited surface dyslexia (‘pynt’ for pint and ‘soo’ for sew). Anterograde and retrograde memory was impaired. There was a discrepancy in her fluency scores, with a lower score for category than for letter verbal fluency.

She could follow two-stage commands, repeat words and phrases and write in full sentences, such as ‘In my last week holiday, I prayed, sang and worshipped the lord, through my lord Jesus Christ’ and ‘I watched TV and did not fully enjoy what I saw’.

Her object naming was poor, with superior performance for high-frequency inanimate objects (she correctly named the spoon and book). Although she was unable to identify low-frequency animals such as the kangaroo, she had retained semantic knowledge in that she said ‘it jumps’. There was evidence of loss of fine-grained semantic knowledge, with retained superordinate category knowledge identifying a penguin only as a ‘bird’.

The case is summarised in Box 1 and key questions are presented in Box 2.

Box 1 Case summary

- GP referral: 69-year-old West African woman, hyperreligious, responding to unseen stimuli
- In clinic: fixed on religious proclamations alongside grandiose and paranoid beliefs, with poor recollection of early life events
- Collateral: religiosity increased over the years, associated with congruent behavioural abnormalities, misuse of household objects and social disinhibition
- Cognition: surface dyslexia and impairment of anterograde and retrograde memory, fluency and object naming

Box 2 Questions to address

- What could be the differential diagnosis in this situation?
- How can neurodegeneration be differentiated from a primary psychiatric disorder?
- What further tests and investigations are required?
- What management is appropriate?
- What is the relevance to hyperreligiosity?

## Diagnosis

**Fig. 1:**
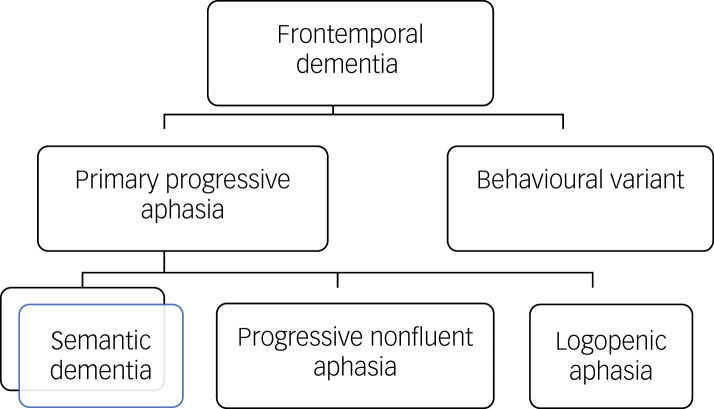
The subtypes of frontotemporal dementia.

Primary progressive aphasias (PPAs) refer to neurodegenerative syndromes where there are early and prominent effects on the domain of language. There are three main presentations, including semantic dementia (Fig. 1).^2^ For semantic dementia the presentation reflects a progressive loss of semantic knowledge or ‘learned knowledge about the world’.

Arnold Pick had described a progressive disorder of language with associated frontotemporal atrophy in the late 19th century; however, it was not until 1975 that Elizabeth Warrington wrote about a selective impairment of semantic memory in three individuals with anomia.^3^ In the same decade, Tulving had conceptualised memory into semantic and episodic systems^4^ and then the first cases were described by Snowden et al (1989)^5^ and Hodges et al (1992),^6^ coining the term ‘semantic dementia’.

Semantic memory encompasses not just our knowledge of words and their meanings, but also learned properties of objects and more abstract conceptual knowledge, including moral and societal rules.^7^ This understanding underlies our fundamental ability to navigate and interpret the world around us.^8^

People with neurodegeneration of the anterior temporal lobe (ATL) demonstrate not only verbal but also non-verbal semantic impairments, which leads to the conclusion that this region represents supramodal semantic knowledge. Building on this, Zahn et al showed, using functional magnetic resonance imaging (fMRI), how the ATL represents abstract social semantic knowledge.^9^

### Diagnostic criteria

The diagnosis of semantic dementia is clinical and is dependent on a formal cognitive assessment. The core diagnostic criteria are impairment of the following.

- *Confrontation naming*: Tested by object naming in the Addenbrooke's Cognitive Assessment (ACE-III). It requires engagement of semantic memory and access to the mental lexicon (store of words). A familiarity effect is often seen, where more commonly used or high-frequency words such as ‘book’ are remembered whereas lower-frequency words such as ‘accordion’ are not.^3^ Notably, this test may also be confounded by other conditions affecting visual perception and word retrieval (such as Alzheimer's disease). A useful differentiation can be if you provide a cue to assist retrieval (e.g. the first letter or syllable) or a choice of the target word from three: a person with Alzheimer's disease may be able to capitalise on this to identify the word whereas someone with semantic dementia will not.
- *Single-word comprehension*: When asked ‘What do we call a small seat without a back’, the patient replied ‘a table’. The steady loss of the referential meaning of words (seen or heard) impairs comprehension, which is dependent on lexico-semantic processing.^10^ This deficit underlies characteristic speech deficits in semantic dementia, namely circumlocution, semantic paraphasia (saying ‘car’ instead of ‘drive’) and superordinate responses (‘instrument’ instead of ‘harp’).^11^

The supportive diagnostic criteria emphasise ruling out the other primary progressive aphasias: logopenic progressive aphasia (LPA) typically has impaired sentence repetition, with a length-dependent effect as the disease progresses, whereas with progressive non-fluent aphasia (PNFA) the sentence is halting and effortful, with agrammatism and telegraphic speech.^2^

### Epidemiology

The estimated prevalence of FTD is 10.8/100 000, with semantic dementia accounting for around one-third of cases.^12,13^ There is a wide age range at presentation (40–79 years), with a mean age at diagnosis of 64.2 years. Progression is usually slower than in other forms of FTD, with a 50% survival at 12.8 years.^14^

### Neuropathology

The variants of FTD all involve a frontotemporal network-based, prion-like spread of a misfolded protein.^15^ In semantic dementia, most cases are caused by a mutant version of a transcriptional repressor called Tar DNA Bind Protein-43 (TDP-43) Type C. This differs from behavioural variant FTD (bvFTD), which has less predictable pathology as it can be caused by mutant TDP-43, but also by tau and fused in sarcoma (FUS) protein (in order of decreasing pathological frequency).^16^

### Progression of language, memory and behavioural disturbance

The course of the disease is gradual, beginning with naming difficulties. As the semantic store is eroded, so too is the ability to discriminate between related concepts. Over time, other non-language domains are affected, including the recognition of voice, tactile stimuli and knowledge of object use.^17^ Pathology is initially limited to the left temporal lobe, but for right lateralised patients (roughly 30%), rather than an initial loss of semantic knowledge, there may be a loss of ‘person knowledge’ entailing prosopagnosia and a decline in social cognition.^18^

In semantic dementia there can initially be a reverse temporal gradient of memory loss, where memory is poorer for remote rather than recent events. This likely represents the semanticisation of information, as memories for events (pinpointed in time and place) move from the relatively preserved episodic store into the semantic system.^19,20^

The impact on quality of life (QoL) for both the patient and their carers gradually intensifies as a behavioural syndrome emerges of mental rigidity, inflexible behaviour, obsessionalism and altered food preferences. Additional symptoms such as apathy and impaired theory of mind develop, more typically associated with bvFTD.^21^ Ultimately there is a complete loss of communicative language, leaving only stereotyped phrases or even mutism.^22^

## The differential diagnoses

The clinical assessment of semantic dementia requires differentiation from other neurodegenerative conditions, such as bvFTD and Alzheimer's disease.

- *bvFTD:* The Rascovsky criteria outline the six features that carry consensus for the diagnosis.^23^ In this case the patient's son described personality and behaviour changes, as well as dietary changes. However, over time, many patients with a language variant of FTD go on to develop behavioural symptoms that typify bvFTD.^24^ Key point: the focus in history taking should therefore be on the earliest or first presenting features for diagnostic categorisation.^23^
- *Amnestic Alzheimer's disease*: Features suggestive of amnestic Alzheimer's disease include episodic memory impairment and anomia. Semantic paraphasia can also feature in moderate Alzheimer's disease. An atypical form of Alzheimer's disease, frontal variant Alzheimer's disease, should also be considered. It involves executive dysfunction with progressive behavioural change, predominating and pre-dating an episodic amnesia.^25^ The severe semantic memory deficit in this patient made frontal variant Alzheimer's disease unlikely. In true cases of diagnostic uncertainty additional investigations such as cerebrospinal fluid biomarkers or fluorodeoxyglucose positron emission tomography (FDG-PET) may be used to help differentiate, particularly in younger patients.

## Neurodegeneration versus a primary psychiatric disorder

Psychosis in older age can be part of a dementia, delirium or schizophrenia-like condition (psychotic depression with pseudodementia requires strong consideration). Although the onset of schizophrenia most commonly occurs in late adolescence or early adult life, there is a variant of non-affective functional psychosis that can occur after age 60 called very late-onset schizophrenia-like psychosis (VLOSLP).^26^

The common characteristics of VLOSLP (which help differentiate from early-onset psychosis) are:

- persecutory delusions relating to spying or home invasion by neighbours
- partition delusions (a belief centred on the permeability of a barrier such as wall)
- visual and tactile hallucinations are more common, whereas formal thought disorder is less common^27^
- importantly, the degree of cognitive impairment is not significantly higher than in those with early-onset psychosis.^28^

Delusions are common in neurodegenerative disorders too; in the largest series of 97 neuropathologically confirmed cases of FTD, 32% had psychotic symptoms, 20.6% had paranoid ideas, 17.5% hallucinated and 17.5% had delusions, with psychotic symptoms present in all pathological subtypes (tau, TDP, FUS).^29^ Delusions were most commonly paranoid or persecutory, followed by erotomania.^29,30^

In Alzheimer's disease approximately 50% of patients experience delusions and around 30% experience hallucinations.^30^ Studies using the informant-based Behavioral Pathology in Alzheimer's Disease Rating Scale (BEHAVE-AD) have reported that delusions of theft, abandonment and one's house not being one's home are often seen in Alzheimer's disease.^31^

In Lewy body dementia (LBD) a spectrum of psychosis exists, where delusions, illusions and hallucinations become more elaborate and pronounced as cognitive impairment and insight worsen.^32^ Delusions in LBD are more common than in Alzheimer's disease and tend to exist alongside visual misperceptions. Common delusions include Capgras phenomenon (believing a familiar person is an imposter), Othello delusion (believing that a spouse or partner is unfaithful), reduplicative phenomena (recognising a familiar environment as looking the same, but being an imitation/copy) or reference from the television.^33^

## Further tests and investigations

Neuroimaging displaying marked anterior temporal lobe (ATL) atrophy can be a diagnostic keystone and pathognomic,^34^ as the molecular pathology is highly conserved in the presence of this imaging finding. Within the ATL there is a predilection for superior temporal gyrus atrophy and in 30% of cases right lateralised ATL atrophy occurs.^2,18^ Right hemispheric cortical involvement becomes more pronounced with disease progression if it is not apparent from the onset.

**Fig. 2:**
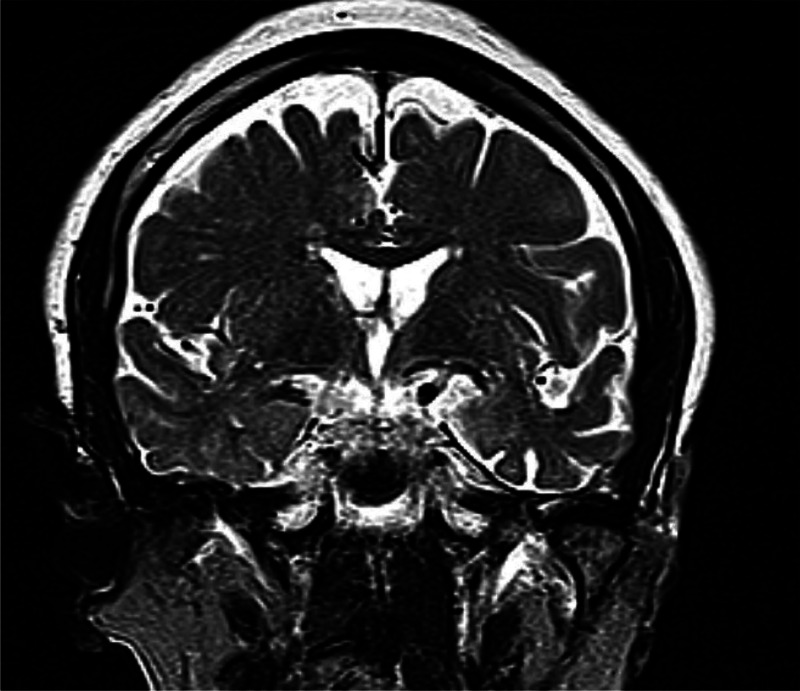
T_2_-weighted axial image displaying the left-side anterior temporal atrophy.

This patient's MRI (Fig. 2) showed a focal right ATL atrophy *without* disproportional hippocampal atrophy. Where there is diagnostic uncertainty, NICE also recommend the use of FDG-PET and perfusion SPECT.^35^

In semantic dementia there is a strong clinicopathological concordance and genetic testing is not as relevant as in other FTD syndromes. Unlike other forms of FTD, semantic dementia is often sporadic and non-familial, and potentially due to post-zygotic (after fertilisation) or late-somatic variants during brain development.^36^

## Management

Currently there are no disease-modifying treatments for any of the FTD disorders, although there are trials underway investigating treatments for genetic forms of FTD. The Genetic Frontotemporal Dementia Initiative (GENFI) is a group of research centres across Europe and Canada investigating genetic forms of FTD, focusing on mutations in the *GRN*, microtubule-associated protein tau (*MAPT*) and *C9orf72* genes. They are currently in their third phase, aiming to prepare GENFI participants for therapeutic trials with pharmaceutical companies.^37^

At present, management revolves around caregiver education, environmental modification and working with speech and language therapy to optimise communication through aids. Two commonly encountered issues are:

- pharmacological management of neuropsychiatric symptoms
- availability of non-pharmacological interventions and specialist therapeutic input.

### Pharmacological approach

The current role of medication is to address the neuropsychiatric manifestations such as depression, apathy, anxiety, delusions and obsessionalism. There is a shortage of good clinical trials looking at the treatment of psychotic symptoms in people with dementia, and of those that do, there is a focus on Alzheimer's disease and LBD.^38^

Although the antipsychotic-associated risks are well-known (cerebrovascular events, increased mortality and Parkinsonism),^39^ the patient factors associated with increased risk of stroke are less well researched. Risperidone is commonly used in Alzheimer's disease and FTD and was recently reviewed as regards patient factors associated with better or worse outcomes. Better outcomes were associated with baseline features of depression and delusions, whereas worse outcomes were associated with concurrent prescription of non-steroidal anti-inflammatory drugs (NSAIDs).^40^

A thorough medication review is important, with a particular focus on medications with a high anticholinergic burden (such as promethazine and hyoscine) to avoid iatrogenic worsening of cognition. Cholinesterase inhibitors and memantine may worsen behavioural symptoms, with the possible exception of rivastigmine.^41,42^

The underlying neurotransmitter deficits in semantic dementia are broad; however, particular focus has been paid to serotonin,^43^ which regulates higher brain functions related to cognitive control, learning and affect, while modulating synaptic plasticity. Its downstream effects include modulating other neurotransmitters (e.g. inhibiting glutamate release in the frontal cortex).^44^ In FTD, 5-HT_1A_ and 5-HT_2A_ receptor density is reduced in the hypothalamus and frontotemporal region. Most of the studies looking at boosting serotonergic transmission (with selective serotonin reuptake inhibitors (SSRIs) or trazadone) are small, uncontrolled and of short duration, and show mixed results.

One double-blind placebo-controlled trial looking at paroxetine in FTD found no improvement, but rather a selective impairment in cognition,^45^ which may relate to its strong anticholinergic effects. Another double-blind placebo-controlled study looking at trazadone found a significant decrease in the Neuropsychiatry Inventory (NPI) score compared with placebo, largely in agitation and eating disorders.^46^ Trazadone's mechanism is different from SSRIs as it *antagonises* a range of serotonin receptors (apart from 5HT_1A_). Although it is often said that there is a serotonin deficiency in FTD, this may be an oversimplification, especially as a post-mortem study found an extraneuronal excess of serotonin (and a decrease in its metabolites).^47^ It is more likely that serotonergic transmission and metabolism is affected, with downstream effects on other neurotransmitters (such as on frontal glutamate release).

There are no high-quality trials of antipsychotic drugs in FTD, but the use of atypical antipsychotics such as risperidone or quetiapine may be considered when neurobehavioural risks outweigh those associated with side-effects and increased mortality.

### Non-pharmacological approach

Non-pharmacological interventions are the most important and effective treatments currently available. The overarching principles in the management of semantic dementia are: psychoeducation, early referral to a specialist and the involvement of the multidisciplinary team (MDT). Certain challenges can be predicted by understanding the course of the disease, for example:

- the loss of semantic knowledge and subsequent failure to recognise everyday objects may result in the misuse of household items and potential harm
- declining social cognition and prosopagnosia may increase risk and carer distress.

NICE underscores the importance of signposting patients and their caregivers to either a primary care or hospital-based dementia service.^35^ Crucial too is the allocation of a named care coordinator, from health or social care, who may help navigate the patient and their carer/family to resources and therapies. In this case, the patient was referred to the National Hospital for Neurology and Neurosurgery (NHNN) cognitive disorders clinic, and she was directed to its affiliated Rare Dementia Support (RDS) website, where there are resources dedicated to community, learning and advice for people with primary progressive aphasia.^48^

For people who are not based in London, their local memory clinic/cognitive disorders centre should be involved, but the RDS also has a calendar of regional meetings for further support. The team at the Cerebral Function Unit (a cognitive neurology clinic based at Salford Royal Hospital) noticed a gap in support for carers of people with non-Alzheimer's related dementia in the North of England. In 2004 they set up the Carers Support Group and hold quarterly meetings where they provide advice and social support in an informal setting, with social and legal professionals present.

Speech and language therapy (SLT) plays a crucial role in management; therapists can assist in individualising training interventions, communication aids and compensatory strategies to support ADLs.

Box 3 describes a family's experience of semantic dementia.

Box 3 A family's experience of semantic dementia

Kindell et al published the first piece of qualitative research on a family's experience of semantic dementia.^56^ The patient's frustrations lay in difficulty recalling names of people, places and objects. Routines and rituals emerged, such as making regular trips to the same shop to buy cake, rubbing objects (his hands, soles of his shoes, glasses), sorting through rubbish bins and wearing the same clothes. His family became accustomed to his routines over time; rather than trying to change them they incorporated them into their care. Conversational routines grew too, where he would primarily focus on the Second World War and would overuse certain words or phrases. His personality changed to one that seemed more sociable and jovial, as he would stop and talk to strangers, often using overfamiliar language (which his family needed to monitor). The authors highlight the juxtaposition of his abilities (e.g. navigating the neighbourhood) with his disabilities (e.g. inability to use a comb). The impact on spontaneity within the family's life also stood out, with a growing focus on behavioural and conversational routines, and a loss of simple questions like ‘how are you?’.

## Hyperreligiosity in dementia

Hyperreligious patients require a holistic approach, considering both cultural and neuropsychiatric factors. The literature differentiates between an intensification of religious beliefs and ecstatic experience. The former is more commonly seen in FTD (predominantly affecting the temporal lobes) than in Alzheimer's disease or DLB.^49,50^ One's sense of self is also important, influenced by: semantic information (knowledge about one's personal attributes), autobiographical memories (often affect laden) and will (motivation to maintain one's prior beliefs).^51^ Furthermore, alteration in the integrity of self versus other in the right cultural context is thought to manifest as hyperreligiosity or a transcendent meditative experience.^52^

These attributes have strong frontotemporal correlates, particularly with the prefrontal regions.^53^ There may be a role played by overactive ventromedial dopaminergic systems, resulting in the transfer of attention and goal-directed behaviour to extra-personal space.^52^ Given that some people with FTD show an obsessional interest in traditionally rewarding stimuli such as sweet food or even music (musicophilia), FTD can be thought of as a disease that alters reward sensitivity and therefore reward-seeking behaviours.^54,55^ In addition, the more common behaviours in FTD, namely impulsivity, delusions and mental rigidity, may serve to reinforce the hyperreligiosity and worship behaviour.

## Conclusions

The diagnosis of semantic dementia can be straightforward in terms of its clinicopathological correlation and signature cognitive deficits, but it can masquerade as a primary psychiatric disorder with its neuropsychiatric manifestations. Patients’ experience of their symptoms can be heavily modulated by cultural and religious beliefs. In this sense, the predominant reported symptoms in patients with semantic dementia may not always be word-finding difficulties, which is why the key is in judicious history taking and careful examination.

A holistic approach in diagnosis is also important for management, and signposting to online and region-specific support can reduce the sense of helplessness felt by patients and carers in rarer disorders. As molecular-targeted treatments begin to emerge, the challenge will be in careful patient selection early in the disease course to identify those who will benefit from treatment. This patient selection, at present, continues to rely largely on clinical skills.

## About the authors

**Richard H. Cole** is a Clinical Research Fellow and PhD student at the Institute of Psychiatry, Psychology & Neuroscience, King's College London, UK and has completed core training with Camden and Islington NHS Foundation Trust, London, UK. **Camilla N. Clark** is an NIHR clinical lecturer in the Institute of Molecular & Clinical Sciences, St George's University of London, London, UK. **Norman A. Poole** is a consultant neuropsychiatrist at in the Department of Neuropsychiatry at St George's Hospital, South West London and St George's Mental Health NHS Trust, London, UK.

## Data Availability

Data availability is not applicable to this article as no new data were created or analysed in this study.
